# Editorial: Established and Novel Roles of Platelets in Health and Disease

**DOI:** 10.3389/fcvm.2022.835615

**Published:** 2022-01-31

**Authors:** Paul Jurasz, Vera Ignjatovic, Marie Lordkipanidzé

**Affiliations:** ^1^Faculty of Pharmacy and Pharmaceutical Sciences, University of Alberta, Edmonton, AB, Canada; ^2^Department of Pharmacology, Faculty of Medicine and Dentistry, University of Alberta, Edmonton, AB, Canada; ^3^Cardiovascular Research Centre, University of Alberta, Edmonton, AB, Canada; ^4^Mazankowski Alberta Heart Institute, University of Alberta, Edmonton, AB, Canada; ^5^Department of Haematology Research, Murdoch Children's Research Institute, Melbourne, VIC, Australia; ^6^Department of Paediatrics, The University of Melbourne, Melbourne, VIC, Australia; ^7^Research Center, Montreal Heart Institute, Montreal, QC, Canada; ^8^Faculty of Pharmacy, Université de Montréal, Montreal, QC, Canada

**Keywords:** platelets, thrombosis and haemostasis, non-traditional roles, special populations, platelet function

Platelets have been long recognized for their role in maintaining hemostasis, with defects in their number or function leading to bleeding. This has led many to simply think of platelets as “band-aids” of the vascular system (1). Conversely, as appreciated by cardiologists, neurologists, and hematologists alike, platelet hyperactivity is itself a predisposition to thrombosis. Beyond the recognized Yin and Yang dualism of regulating bleeding and thrombosis, platelets are increasingly emerging as key regulators of an array of diverse physiological and pathological processes. Technological advancements have allowed researchers to identify the contribution of platelets to vascular and lymphatic development (2–4), maintenance of vascular integrity and maturation of the circulatory system (5, 6), as well in formation of new blood vessels (7–9). The fact that platelets also participate in inflammation and immunity, in part, via their surface expression of P-selectin, toll-like receptors (10, 11), complement receptors (12), CD40L (13), and programmed death—ligand 1 (14) is now well recognized. Correspondingly, platelets respond to both viral and bacterial infections (15–17), and have even been caught in the act of migration to scavenge bacteria (18). Platelets also contribute to wound healing and bone formation (19). Thus, platelets and their-derived products are also frequently used as therapeutic agents, increasing the healing efficiency in maxillo-facial and plastic surgery (20), as well as in sports medicine (21).

On the other hand, platelets are involved in pathological conditions including but not limited to atherosclerosis and related diseases, cancer (22), and diseases of the central nervous system including Alzheimer's disease, depression and multiple sclerosis (23–25). Moreover, platelets participate in the pathophysiology of auto-immune diseases, such as allergies, skin diseases, rheumatoid arthritis and liver disease (26–28).

This extraordinary capability of platelets to regulate such an extensive array of physiological and pathophysiological processes largely stems from their ability to store and release a wide range of biologically active substances via their granules and microparticles (29, 30). Platelets also express biochemical and functional heterogeneity, with numerous platelet subpopulations having been identified, including the procoaguant platelet type (31, 32). This potential specialization of function is likely to contribute to the diverse roles of platelets. It is therefore unsurprising that platelets are no longer viewed as simply “band-aids” of the vascular system. In an effort to raise awareness of the ever-increasing scope of platelet functions, this Research Topic aims to increase our current understanding of the wider contribution of platelets in physiological and pathological conditions.

Identifying novel genetic determinants of platelet disorders, Almazni et al. summarize advancements in diagnosis of inherited thrombocytopenia, covering the spectrum from phenotypic approaches, high-throughput sequencing, to targeted panels and bioinformatics. The authors conclude that a combination of the genomics approaches with high throughput data handling pipelines is essential in ensuring transformational advancements in patient diagnosis and personalized care.

Rana et al. review our current knowledge of how shear forces impact aggregation and thrombosis. With a major focus on the shear-sensitive protein von Willebrand Factor, the shear-induced activation, conformational changes, interaction with GPIbα and role in platelet arrest is summarized. The authors also discuss platelet aggregation under shear gradients and novel therapeutic strategies that aim to reduce shear-induced platelet activation and thrombus formation.

Kim and Conway review the complement system and describe its interaction with platelets and contribution to early-stage atheroma formation. They describe how complement proteins activate platelets and that, in turn, activated platelets in conjunction with complement proteins may propagate vascular inflammation. Understanding the complex cross-talk between platelet, vascular endothelium, and the complement system in early atheroma formation may allow for development of novel anti-complement therapies to treat atherosclerosis.

In their article Melchinger et al. discuss how a lack of nucleus allows platelets to efficiently travel through small blood vessels and rapidly undergo extreme morphological changes that enable to platelets to efficiently respond to disruptions in hemostasis, infection, and inflammation. Hence, in the absence of a nucleus, the authors describe how mitochondria are key to powering these platelet responses and as well as regulating platelet lifespan.

The ability of platelets to interact with vascular cells and leukocytes at sites of vascular injury and inflammation carves out an important role for platelets as modulators of vascular diseases. In their review, Héloïse Lebas et al. describe how this crosstalk influences vascular diseases in different vascular beds. In their review of the platelet-neutrophil crosstalk, Zucoloto and Jenne explore in more detail how neutrophil extracellular trap (NET)-driven coagulation influences outcomes in the context of infectious diseases, and propose targeting of NETs as a potential therapeutic avenue to uncouple immunity and coagulation. Conversely, this ability of platelets to interact with circulating elements can also be deleterious, an aspect that is discussed by Hante et al. in their review of metal-based nanoparticles developed for medical applications.

Platelet subpopulations are an important area of research. For instance, the ability of platelets to engage with the coagulation system is explored by Reddy and Rand in their review of procoagulant platelets. Phosphatidylserine (PS) exposure, a key event in the platelet-coagulation interplay, is dissected from the perspective of inherited and acquired bleeding disorders, as well as a possible antithrombotic target. Lesyk and Jurasz further dive into platelet subpopulation diversity focusing on their physical, biochemical, and functional heterogeneity, the dynamic nature of platelet characteristics in health and disease, and how differences between individuals can influence platelet responsiveness to antiplatelet therapy. In their review, Le Blanc and Lordkipanidzé look at how age-related changes in platelet biology could lead to thrombotic disease. In the context of global aging populations, it is important to investigate how platelets from older subjects differ in their function and structure from their younger counterparts. Evidence showing platelets from the elderly to be more prone to activation and less sensitive to inhibition highlight important areas for future work.

Ostrowska et al. discuss the current role and future perspectives of platelet function testing to optimize antiplatelet therapy. With evidence of high on-treatment platelet reactivity being associated with thrombotic outcomes and low on-treatment platelet reactivity being associated with bleeding, the idea of a therapeutic window for optimal platelet inhibition is presented. They further highlight that de-escalation of antiplatelet therapies may help balance the delicate bleeding/thrombotic risk. Targeting circadian rhythms in cardiovascular disease to improve the efficacy of antiplatelet agents is at the heart of the review by Buurma et al. The evidence for chronotherapy, especially with aspirin, is presented and discussed, and the authors call for randomized controlled trials with cardiovascular events and side-effects as endpoints, to help translate pharmacological findings to potential clinical benefits.

Extracorporeal membrane oxygenation (ECMO) presents important hemostatic challenges, in already vulnerable patients. Balle et al. summarize the state of knowledge in the setting of adult ECMO patients, and identify a significant knowledge gap in our understanding of platelet function in this critical clinical setting. Specifically, the authors point out the limitations of methods such as platelet aggregation in the setting of low platelet count, as well as in the lack of studies focusing on association between platelet function and clinical outcomes such as bleeding and thrombosis. This is clearly a field with excellent potential for future research, particularly due to the fact that ECMO is a high complication, high cost setting. In addition, the pediatric population is often under-studied, and the very limited platelet phenotype and function data in the pediatric ECMO population is further explored by Yaw et al. The systematic review highlights multiple research gaps in the setting of pediatric ECMO, highlighting the need for robust, well-designed studies in children.

In a different register, the review by Al-Hamed et al. provides an overview of platelet concentrates (PC) and the ability of platelets to enhance bone and soft tissue healing. Subsequently, the authors summarize the various clinical applications of platelet concentrates in the context of oral and craniofacial regeneration, and discuss some of the reasons for the inconsistencies in observed effects of PC on tissue regeneration.

This Research Topic also contains four original research articles. Using a standardized microfluidics and multi-parameter approach Nagy et al. quantitatively examine the changes in collagen-dependent thrombus formation for 38 different strains of genetically modified mice. Their novel approach can be used to determine the severity of the platelet defect and stage at which the altered thrombus function occurs. Diallo et al. investigate the impact of platelet pathogen reduction technologies on platelet microRNA content demonstrating that they may impact microRNA loading into platelet microparticles. Balle et al. present an original prospective study of platelet function in 33 adult patients undergoing ECMO support utilizing impedance aggregometry and flow cytometry. The authors present a novel approach to data analysis and interpretation by accounting for platelet count when interpreting the aggregometry data, and suggest that platelets might not be as impaired during ECMO support as previously suggested. This study confirms the need for additional studies in this field. Rodriguez and Johnson show platelet parameters that segregate with diabetic status, using platelet function assessment collected in the Framingham Heart Study Offspring cohort and platelet indices available in the UK BioBank cohort. The largest analysis to date, this study argues for the use of mean platelet volume as a biomarker to segment pre-diabetics and diabetics for risk prediction.

We hope that the articles within this Research Topic will spur further research into the diverse roles platelets play in physiological and pathological conditions within and beyond hemostasis and thrombosis.

## Author Contributions

All authors listed have made a substantial, direct, and intellectual contribution to the work and approved it for publication.

## Conflict of Interest

The authors declare that the research was conducted in the absence of any commercial or financial relationships that could be construed as a potential conflict of interest.

## Publisher's Note

All claims expressed in this article are solely those of the authors and do not necessarily represent those of their affiliated organizations, or those of the publisher, the editors and the reviewers. Any product that may be evaluated in this article, or claim that may be made by its manufacturer, is not guaranteed or endorsed by the publisher.
